# Lactic Fermentation as a Strategy to Improve the Nutritional and Functional Values of Pseudocereals

**DOI:** 10.3389/fnut.2019.00098

**Published:** 2019-07-03

**Authors:** Graciela C. Rollán, Carla L. Gerez, Jean G. LeBlanc

**Affiliations:** Centro de Referencia para Lactobacilos (CERELA) - CONICET, San Miguel de Tucumán, Argentina

**Keywords:** lactic acid bacteria, pseudocereals, vitamins, phytate, phytochemicals

## Abstract

One of the greatest challenges is to reduce malnutrition worldwide while promoting sustainable agricultural and food systems. This is a daunting task due to the constant growth of the population and the increasing demands by consumers for functional foods with higher nutritional values. Cereal grains are the most important dietary energy source globally; wheat, rice, and maize currently provide about half of the dietary energy source of humankind. In addition, the increase of celiac patients worldwide has motivated the development of gluten-free foods using alternative flour types to wheat such as rice, corn, cassava, soybean, and pseudocereals (amaranth, quinoa, and buckwheat). Amaranth and quinoa have been cultivated since ancient times and were two of the major crops of the Pre-Colombian cultures in Latin- America. In recent years and due to their well-known high nutritional value and potential health benefits, these pseudocereals have received much attention as ideal candidates for gluten-free products. The importance of exploiting these grains for the elaboration of healthy and nutritious foods has forced food producers to develop novel adequate strategies for their processing. Fermentation is one of the most antique and economical methods of producing and preserving foods and can be easily employed for cereal processing. The nutritional and functional quality of pseudocereals can be improved by fermentation using Lactic Acid Bacteria (LAB). This review provides an overview on pseudocereal fermentation by LAB emphasizing the capacity of these bacteria to decrease antinutritional factors such as phytic acid, increase the functional value of phytochemicals such as phenolic compounds, and produce nutritional ingredients such as B-group vitamins. The numerous beneficial effects of lactic fermentation of pseudocereals can be exploited to design novel and healthier foods or grain ingredients destined to general population and especially to patients with coeliac disease.

## Introduction

According to the Food and Agriculture Organization (FAO), global hunger is in ascent again after constantly decreasing for over a decade (1). The number of chronically undernourished people in the world is estimated to have increased to 815 million (11% of the global population) in 2016, up from 777 million in 2015, as reported in the edition of the Annual United Nations on World Food Security and Nutrition published in September 2017, based on reports by five organizations [ONU, FAO (Food and Agriculture Organization), WHO (World Health Organization), IFAD (International Fund for Agricultural Development (IFAD), and World Food Program (WFP)] (1). At the same time, different forms of malnutrition are threatening the health of millions of people worldwide. Nearly 795 million people have eating disorders and do not carry out healthy and active lifestyles with an estimated 41 million children that are now overweight according to the World Food Program. Added to this serious situation, the world population is expected to reach nine billion persons in the coming decades, imposing the need for urgent solutions to increase food supplies (2). In addition, climate change is rapidly degrading the conditions of crop production, affecting the availability of water and arable land, increasing salinization and aridity, generating a serious problem in the yield of food. It is estimated that approximately one billion hectares or crop land will be affected worldwide due to these problems, especially those in the hottest and most arid regions of the world (3–7). In addition to climate change, global staple crop production is also threatened by restrictions such as accelerated erosion of soil and natural resources (8). Frison et al. (9) also reported that modern agriculture generates serious problems in the environment causing soil degradation and erosion, water pollution and biodiversity decline. Therefore, it is essential to increase food production for a growing population that uses low input regimes. The FAO urges to expand the response to climate change in agriculture. According to their 2017 document “A systemic approach that involves the relevant agricultural and food sectors and those interested in the adoption of agroecology, has the potential to greatly accelerate the transition to sustainable and resilient food systems, in line with the various international commitments assumed by the member countries.” Agroecology, in an integral manner, can support the execution of different social, economic, environmental, nutritional, and health objectives.

Diets throughout the world are based on two dozen crops with a dominant proportion of the “big three” cereals: wheat (*Triticum aestivum*), maize *(Zea mays*), and rice (*Oryza sativa*), which contribute to approximately 60% of the total caloric intake (10). However, these crops may not intrinsically be the best-suited species to face up to extreme weather events that are becoming more frequent due to climate change; thus, world grain production per capita is expected to decline by at least 14% between 2008 and 2030 (11). The rapid growth of the world population and per capita food consumption worldwide puts great pressure on the food industry to produce more food (12). The food supply must double by 2050 to counterbalance the effects of climate change and population pressure on global food systems and thus novel food sources must be found (6). Less than 0.6% of plant species that are suitable for human consumption have reached the world markets (13). The diversification of main crops and the systems in which they grow is essential for agriculture to be sustainable, resilient, and suitable for local environments and soils in the future. One critical measure to ensure future food availability for all is to provide more diverse food sources and develop agricultural systems that are resistant to climate change. Furthermore, the new challenge for the food industries and scientific areas such as chemistry, biology, medicine, pharmacology, and food technology is to obtain foods with a higher nutritional value that also possess functional properties which go beyond traditional health requirements. In response to this issue, one of the leading strategies is unlocking the potential of underutilized crops. Most of these crops have high nutritional value, resilience traits, with the ability to withstand drought, flooding, extreme temperatures, and pests and diseases better than current major staples and thus they should be investigated, developed, and now more than ever used (14). Current emphasis is now placed on the use of ancient cereals and pseudocereals that include amaranth, buckwheat, quinoa, teff, millets amongst others.

The aim of this review is to highlight certain nutritional and functional properties of pseudocereals and how lactic acid fermentation can be used as an advantageous biotechnological strategy to improve the natural potential of these grains. This review provides an overview on pseudocereal fermentation by lactic acid bacteria (LAB) emphasizing the capacity of these bacteria to decrease antinutritional factors such as phytic acid, increase the functional value of phytochemicals such as phenolic compounds, and produce nutritional ingredients such as B-group vitamins.

## Crop Diversification: Pseudocereals

Crop diversification is an important strategy to protect global food supplies and to fight against malnutrition. Sustainable diets should provide nutritious food at affordable costs, while having a low impact on the environment (15). Effective analysis of sustainable plant resources is an important assignment for ensuring global food security in the future (16).

The need for the diversification of grains for human consumption and the consumer's demands for gluten-free and more nutritious products caused the resurgence and valoration of underutilized crops, so-called minor grains such as sorghum, millets, and pseudocereals through the world during the last several decades (17). In the “International AACC list of recognized grains” pseudocereals are also mentioned (18) where the most important species are quinoa (*Chenopodium quinoa* Willd), amaranth (*Amaranthus* sp.), and buckwheat (*Fagopyrum esculentum*). Pseudocereals are dicotyledonous species unlike true cereals (Poaceae family), that are monocotyledonous species. Pseudocereals are known as such since they are similar to cereals in their physical appearance and their seeds are edible with high starch content that can be milled into flour (19). Their high nutritional value is mostly due to their elevated content and quality of proteins (20, 21).

**Figure 1:**
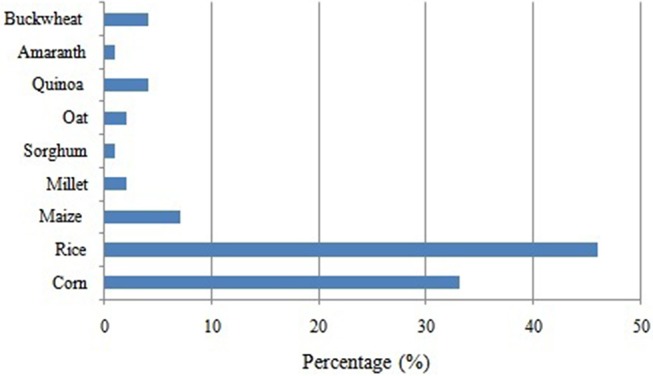
Principal grains used for gluten-free products on the market [adapted from (25)].

Celiac disease (CD) is one of the most common lifelong disorders worldwide with as estimated mean prevalence of 1% of the general population (22). The increase of celiac patients throughout the world has led to intensify the search for alternative flours to wheat (19). The development of gluten-free (GF) products is therefore essential and poses novel challenges for food producers (23). In the last decade, due to the pseudocereals characteristics, GF and good nutritional advantages, the use of these grains has increased for their addition in healthy diets especially for people allergic to cereals. Thus, the integration of these grains into GF diets could be a valuable contribution for improving the quality of the existing GF products, which have been mainly based on rice and maize flour (24). Despite the fact that the interest in pseudocereals due to its high nutritional value has increased, only a few products including these grains are available on the market [Figure 1; (25)].

## Pseudocereals Historical Background

Pseudocereals nourished the Native Americans populations and allowed them to increase their endurance and mental development; because of their properties, Mayans and Incas considered these grains sacred. The conquest of America meant not only a political and social domination of indigenous civilizations, but also a change in their feeding habits. Jacobsen (26) reported that quinoa is one of the oldest crops in the Andean region, having been grown for approximately 7000 years; it is considered the principal crop of the pre-Columbian cultures in Latin America (27, 28). The Incas called quinoa “the mother grain” for many reasons: (i) it is one of the few crops able to grow in high salt soils in Southern Bolivia and Northern Chile, (ii) it has high tolerance to abiotic stresses, (iii) it grows in soils with water scarcity, and (iv) it is resistant to extreme temperatures (−4 to 38°C) and harsh climate conditions (15, 27, 29, 30). The FAO (United Nations) declared, “quinoa has the balance of proteins and nutrients closest to the ideal food for humans.”

Amaranth (*Amaranthus* sp.) is an ancient crop consumed during the Mayan and Aztec periods. It was called “the Inca wheat” by the Spanish conquerors. Amaranth grain species are annual herbaceous plants native of America but they are also now distributed in Asia and Africa (31, 32). *Amaranthus caudatus* was discovered in the north of Argentina (Salta) 2000 years ago (33). When the Spaniards arrived, they decided to exterminate pseudocereals because of their religious implications. Ironically, it is now Europeans who teach us how to consume the grains that were used by the Native American civilizations. The interest in these Andean ancestral crops in the world has led to an increase in their cultivation and production in recent years.

Buckwheat (*Fagopyrum esculentum* Moench) has its origin in Asia and it is believed to have been cultivated in China during the fifth and sixth centuries. It came to Europe after some 800–900 years and to North America in seventeenth century (34).

**Figure 2:**
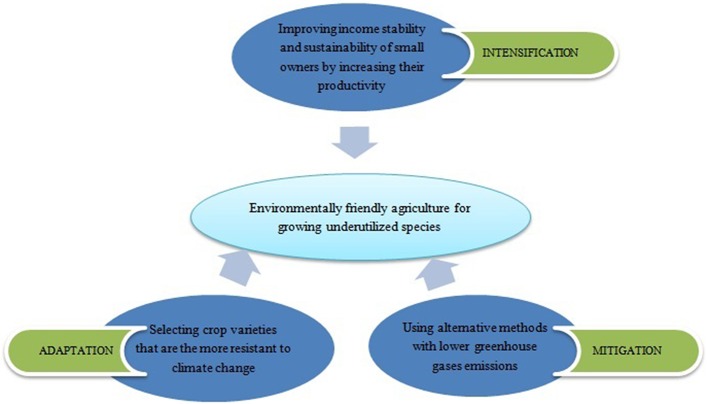
Strategies to use underutilized species and increase the crops biodiversification for a sustainable agriculture [adapted from (35)].

Underutilized species by means of sustainable intensification, adaptation and mitigation can accelerate the process to obtain climate-smart agriculture [Figure 2; (35)].

## Nutritional Value

Charalampopoulos et al. (36) reported that 73% of the total world harvested area corresponds to cereal crops and contributes to more than 60% of the world food production, furnishing proteins, minerals, dietary fiber, and vitamins necessary for human health. Cereals contribute around 50% of the mean daily energy intake in most populations, and 70% in some developing countries, converting them into one of the most important sources of energy in the world (37). However, most grains are, to a greater or lesser extent, deficient in a number of elemental nutrients such as the essential amino acids threonine, lysine, and tryptophan. Their protein digestibility is also lower than that of animal origin, due partially to the presence of phytic acid, tannins, and polyphenols which bind to protein thus making them indigestible (38). Pseudocereals in turn have been described as “the grains of the twenty-first century” (39, 40). The FAO classified quinoa as one of humanity's promising crops destined to contribute to food security in the twenty-first century by its high nutritive potential and genetic diversity [Food and Agriculture Organization Regional Office for Latin America and PROINPA, (27, 41)]. Quinoa and amaranth have tender leaves that are used in food preparation; however, it is their grains that attract the most interest due to their high nutritional value. They are rich in proteins of excellent quality with a balanced essential amino acid composition that include abundant amounts of sulfur- rich amino acids (42). They are also a good source of minerals (calcium, iron and zinc), vitamins, and natural antioxidants (43). They are a significant source of compounds such as flavonoids, polyphenols and phytosterols with potential nutraceutical benefits. From a food provision perspective, pseudocereals are potentially important crops due to their properties (exceptional nutritional value, ability to grow in dry conditions, and their resilience to climatic conditions). Several reviews have reported the nutritional value of Andean grains (19, 43–49).

The General Assembly of the United Nations declared 2013 as the International Year of Quinoa (IYQ), with the goal of focalizing global attention on the role it can perform in contributing to food security, nutrition, and poverty eradication (50, 51). The rapid expansion of the harvested area, with a doubling of countries from 2013, is rapidly changing the perception and representation of quinoa from a minor to a potential major crop. The excellent properties of quinoa led to this ancestral grain to be considered a possible crop in NASA's Controlled Ecological Life Support System for long-duration manned space flights (39).

### Vitamin in Pseudocereals

Vitamins are essential micronutrients since only small quantities are required for adequate growth and function of numerous metabolic reactions. Vitamins are divided based on their solubility in fat (A, D, E, and K), or in water (C and the B-group vitamins). Since there are no foods that contain all vitamins, there is a worldwide increase in their deficiencies due to unbalanced diets. Other causes of vitamin deficiencies are malabsorption that can be due to certain drug treatments or diseases, by the presence of antinutritional factors found in certain foods. Although most vitamins are present in cereals and pseudocereals, a large portion of water-soluble vitamins are lost during processing and cooking, especially when water is used for grain soaking. In this sense, many countries have adopted mandatory fortification programs with specific vitamins and minerals. Folic acid is frequently added in foods of mass consumption (such as different flours) in order to prevent deficiencies in the general population. However, the chemical form of the vitamin used in these programs is controversial. Folic acid, a chemical derivative of folates, is not found in nature and can cause many side effects, especially masking vitamin B_12_ deficiency and affecting the activity of certain liver enzymes, but also has been associated with increased risks of colon and prostate cancers (52). Natural folate, present in numerous different chemical forms in vegetables or produced by certain microorganisms does not cause these undesirable side effects. For this reason, more and more researchers are now searching for more natural methods to increase water soluble vitamins such as folate and riboflavin in foods to not only prevent deficiencies, but also to reduce the use of chemical additives in the food chain.

It was demonstrated that staple foods produced from amaranth contained total folate contents of 35.5 μg/100 g in bread, 36.3 μg/100 g in cookies, and 38.9 μg/100 g in noodles, whereas when wheat was used, breads contained only 12.0 μg/100 g (53). The riboflavin content in amaranth flour is in the range of 0.29–0.32 mg/100 g, which is about 10-fold higher than that of wheat (54). In general, significant reductions of all vitamins take place during processing, which affect the nutritional value of the products (55). The production of fermented food products with high levels of B-group vitamins increases their commercial and nutritional value and eliminates the need for fortification (56).

### Phytochemical Profile of Pseudocereals

Pseudocereals are important phytochemical sources in the diet. Like cereal, these grains contain a great amount of functional phytochemicals including the phenolic compounds (PC) (57–60). The PC constitute a group of secondary metabolites with important functions in cereals and pseudocereals. The chemical structures of these compounds include an aromatic ring with one or more hydroxyl substituents, and vary from simple phenolic molecules to highly polymerised compounds (61). PC are broadly divided in four classes; phenolic acids (benzoic or hydroxycinnamic acid derivatives), flavonoids (flavonols, flavones, isoflavones, flavanones, and anthocyanidins), stilbenes and lignans. In addition to this diversity, polyphenols may be associated with various carbohydrates and organic acids (60). In general, ferulic, *p*-coumaric, caffeic, isoferulic, vanillic, sinapic, *p*-hydroxybenzoic, syringic, and protocatechuic acids are present in all grains; with ferulic acid the most abundant phenolic acid (62, 63). Gorinstein et al. (64, 65) reported a high content of polyphenols, anthocyanins and flavonoids in pseudocereals such as buckwheat, quinoa and amaranth. Likewise, the highest amount of PC was reported in quinoa (490.2 mg/kg DW), slightly lower in amaranth v. Aztek (464 mg/kg DW), and the lowest in amaranth v. Rawa (424.6 mg/kg DW) (66).

PC may provide health benefits to humans since they are associated with a reduced risk of chronic diseases such as anti-allergenic, anti-inflammatory, anti-microbial, antioxidant, anti-thrombotic, cardioprotective, and stimulates insulin secretion in diabetes mellitus type 2 (57, 67–71). The dietary PC contribute to the maintenance of a healthy gut by modulating the gut microbial balance (beneficial bacteria/pathogen bacteria). Metagenomic and metabolomic studies providing more insight into the health effects of PC in humans are needed to understand the dietary PC/gut microbiota relationship and their mechanisms of action. The PC effect on the modulation of the gut ecology and the two-way relationship “polyphenols ↔ microbiota” is currently being studied (72).

The biological effects of PC depend principally on their bioaccessibility (release of the food matrix in an absorbable form during digestion) and bioavailability (absorption and transference to the bloodstream), and both depend on their chemical structure, matrix interactions, antioxidant activity, and food processing (73–79). Natural PC usually occur as glycosides, esters or polymers that have no biological activity (80). Of the total PC intake, only 5–10% is absorbed in the small intestine and the remaining PC (90–95%) accumulate in the large intestinal lumen where they are subjected to the enzymatic activities of the gut microbial community (81). Food technologists need to find the operating conditions to increase bioaccessibility and bioavailability of PC from the matrix. The addition of purified enzymes such as feruloyl and *p*-coumaryl esterases, xylanase, β-glucanase, and α-amylase from natural sources has been proposed to increase the active PC content in cereals [i.e., wheat and rye; (82–84)]. However, these studies in pseudocereals are still missing.

## Antinutritive Factors in Pseudocereals

Grains of cereals, pseudocereals, and legumes are of global importance in the feeding of monogastric animals (humans and domestic animals) since they are a good source of proteins, bioactive compounds and trace elements (85). However, they contain certain antinutrients compounds, such as phytic acid, saponins, tannins, polyphenols, and protease inhibitors (86). In this sense the bioavailability of minerals in whole grain foods is negatively affected by the presence of phytate (87). Since phytate is an antinutritional factor that is found in the highest quantities in pseudocereals and due to its important negative effect on malnutrition, this review will focus on this antinutritive factor.

### Phytates

Phytic acid (PA) (myo-inositol 1,2,3,4,5,6-hexakisphosphate) is an abundant plant constituent, comprising 1–5% (w/w) of legumes, cereals, pseudocereals, oil seeds, pollen and nuts and represents the largest form of phosphorus storage (88, 89). Besides phytate, myo-inositol 1,2,3,4,5 pentaphosphate and myo-inositol 1,2,3,4 tetraphosphates are also present in seeds, but to a much lower extent (<15%) (90).

Phytic acid is negatively charged at physiological pH, which gives it an extraordinary chelating power with affinity for various components present in foods that are positively charged such as minerals and trace elements. The formed complexes are stable, insoluble and difficult to digest at physiological pH, thus decreasing their bioavailability in the human digestive tract (91).

In certain world populations where staples like wheat, maize and rice are the major or the only source of nutrition, PA as antinutritional factor attracts higher attention because the reduced bioavailability of minerals complexed by it can lead to significant deficiencies in humans (92). Also, Arendt et al. (93) reported that gluten free flours/ingredients have variable concentrations of phytate, i.e., rice, 0.12%; pearl millet, 0.25%; amaranth, 0.47%; teff, 0.70%; lupin, 0.77%; corn, 0.92%; oats,1.13%; quinoa, 1.18%; and soybean, 1.33%. Micronutrient deficiencies affect more than half of the world population, especially in developing countries where plants are the major source of food. Thus, improving the nutritional value of such type of food will improve the nutritional status of entire population (94). High content of phytates in the diet, especially of infants, children, elderly, and people in clinical situations, can significantly decrease the retention of calcium, iron and zinc (95). Reddy et al. (96) reported that PA also is present in the diets of non-ruminant animals, representing 50–80% of total phosphorus content in cereal grains and legumes frequently used in livestock animal feeds. However, phytate phosphorus present in food and feed has low bioavailability and is underutilized due to the lack or low levels of gastrointestinal phytases in monogastric animals (swine, poultry, and fishes) (97, 98). In order to meet the phosphorus requirements in these animals, inorganic phosphorus has to be added to the animal feedstuff as an additional nutrient, which in turn increases the feed cost and phosphorus pollution (99, 100). Undigested phytate and unabsorbed inorganic phosphate are excreted to a large extent (70%) and remains in manure and can lead to its accumulation in the soil and waters. This fact can generate the eutrophication of water, a serious phosphorus pollution problem in areas of intensive livestock production. The eutrophication of water surfaces can then generate cyanobacterial blooms, hypoxia and death of aquatic animals and nitrous oxide production, a potential green- house gas producing a severe environmental problem (101).

PA forms a strong complex with some proteins (the free portion of the basic amino acids such as Lys, Arg, His) and resists their proteolysis. PA negatively affects the absorption of proteins present in foods because inhibits enzymes that are necessary to their digestion such as pepsine. In general, the interaction of phytate with protein is dependent on pH (102).

Lee et al. (103) reported that dietary phytate forms complexes with carbohydrates, reducing their solubility and negatively affecting glucose absorption, leading to a decrease in the glycemic index (blood glucose response). In addition, it was postulated that phytate, by complexing with Ca^2+^ ion, inhibits amylase activity (104).

## Functional Foods and Bioactive Compounds

The demand of consumers for healthier foods has led the food industry to formulate new products within the area of so-called functional foods. Functional foods were defined by Bech-Larsen and Grunert (105) as “Foods that may provide health benefits beyond basic nutrition” and “Food similar in appearance to conventional food that is intended to be consumed as part of a normal diet, but has been modified to subserve physiological roles beyond the provision of simple nutrient requirements.” According to these definitions, certain fruits and vegetables, rich in fiber and bioactive phytochemicals, can be considered functional products. Bioactive compounds are phytochemicals present in plants that can promote health but are not essential for life (106). In the last years, cereals have also been explored due to their potential utilization in developing functional foods (107–109). The key bioactive components of whole grain cereals provide health benefits, principally due to their content of flavonoid and dietary fiber. The covalent interactions between these two components increase their individual anti-inflammatory effects and their positive impact on the gut microbiome (67, 69, 110). In addition to their exceptional nutritional value, pseudocereals are characterized for being rich in many “health-promoting” phytochemicals, such as polyphenols and dietary fiber which exhibit anti-oxidant and free-radical scavenging activity (28, 64, 66, 111–115). Amaranth oil has high levels of tocotrienols and squalene, which are involved in the cholesterol metabolism and could play a significant role in lowering the low-density lipoprotein (LDL)—cholesterol in blood (116). Also, dietary fiber and polyphenols intake has been associated with reduced risk for a number of cardiovascular diseases including stroke, hypertension, and heart disease (117, 118). Pasko et al. (119) reported that the supplementation of a fructose-containing diet with quinoa in male Wistar rats reduced serum total cholesterol, triglycerides, glucose, LDL and plasma total protein and suggests the potential ability of this pseudocereal to prevent cardiovascular disease.

Furthermore, Berti et al. (120) reported that good glycemic control is especially important in CD, as there appears to be a higher incidence of type I diabetes among CD patients. Certain studies *in vivo* demonstrated that pseudocereals have hypoglycemic effects, for this reason they have been suggested as an alternative to habitual ingredients in the production of cereal-based GF products with low GI (120–123). Hence, the utilization of pseudocereals has increased not only in special diets for people allergic to cereals, but also as part of healthy diets (65).

## Fermentation

Fermentation is a metabolic process in which carbohydrates are oxidized to liberate energy in the absence of external electron acceptor. This process is one of the oldest and most economical techniques applied in food preservation and processing (124). Fermented foods, produced and consumed since the development of human civilizations, form part of normal human diet (125). The original purpose of fermentation was the preservation effect. Subsequently, with the development of numerous available preservation technologies, plenty of fermented foods were therefore manufactured because of their unique flavors, aromas, and textures that are much appreciated by consumers. The fermentation of cereals plays a vital role in the production of compounds of great influence on the organoleptic characteristics (such as aroma, taste, and texture) and on the improvement of nutritional properties with a final positive impact on human health (126). Microorganisms are found in almost all ecological niches; cereals and pseudocereals are, in general, a good medium for microbial fermentations. They are rich in polysaccharides, which can be used as a source of carbon and energy by microorganisms during fermentation. Besides carbohydrates, they also contain minerals, vitamins, sterols, and other growth factors (127). Fermented products prepared from more common cereals (such as rice, wheat, corn, or sorghum) and pseudocereals are widespread around the world (128–130). In certain developing countries such as Asia and Africa, high consumption of cereals was reported where these grains are mixed with legumes to improve overall protein quality of the final fermented products (131). Cereal and pseudocereal grains normally have an indigenous microbiota composed by molds, LAB, enterobacteria, aerobic spore formers, etc., which compete for nutrients. The type of microbiota present in each fermented food depends on the pH value, water activity, salt concentration, temperature and composition of the food matrix (132).

### Lactic Acid Fermentation

Lactic acid bacteria are Gram positive, non-sporulating, cytochrome deficient, catalase negative, aerotolerant, fastidious, acid-tolerant, and strictly fermentative microorganisms, produce lactic acid as the major metabolic end product of carbohydrate fermentation (133–135). LAB is a heterogeneous group of microorganisms with GRAS (Generally Recognized as Safe) status that have traditionally been associated with food fermentation (136). The effective carbohydrate fermentation coupled to substrate-level phosphorylation is essential characteristic of LAB; the ATP produced is then employed for biosynthetic functions. LAB are generally related with habitats rich in nutrients, for example different foods (milk, beverages, vegetables, meat, cereals); however, some LAB are members of normal flora of the intestine, mouth, and vagina of mammals (137, 138).

Hammes and Ganzle (139) reported that “Sourdough is a leavening agent traditionally obtained through a backslopping procedure, without the addition of starter microorganisms, whose use in bread making has a long history.” Likewise, according to Hammes et al. (140) “The concerted hydrolytic activities of the grain and microorganisms (LAB and yeasts) are the origin of all cereal fermentations and are best represented by the traditional sourdough fermentation.” The application of selected autochthonous LAB to ferment sourdough constitutes an adequate biotechnology to exploit the potential of cereals, non- wheat cereals and pseudocereals in breadmaking (141, 142). This criterion is of great importance when considering the different biochemical, technological, nutritional, and functional characteristics of different flours. The activity of LAB during cereal fermentation is well-documented. A wide variety of metabolites and compounds, such as organic acids, exopolysaccharides (EPS), antimicrobial compounds, and useful enzymes, among others, are produced by LAB (143–151). There are several different ways how the nutritional and functional quality of cereals and pseudocereals could be improved by their fermentation such as: production of bioactive peptides that may stimulate immune system (152); elimination of cereal gluten (153–158); production of gamma-aminobutyric acid (141); increasing total phenolic content and antioxidant capacity (159–162); improving antiproliferative activity (162); decreasing of antinutritional factors, such as phytic acid, tannins and enzyme inhibitors (163–167).

Traditional cereal- and pseudocereal-fermented products are made all over the world, mainly widespread in Asia and Africa (168). Innovative functional fermented foods were formulated using cereal matrices and LAB (169–172). Currently, there are many products derived from cereals fermented by LAB, however only a few are derived from pseudocereals. Fermented quinoa-based beverages were developed by Ludena Urquizo et al. (173) and Jeske et al. (174). Jeske et al. (175), reported the beneficial effect of fermentation by mannitol-producing LAB in combination with various exogenous enzymes in the reduction of sugar in a quinoa-based milk substitute.

#### Improving the Functional Phytochemical Value by Lactic Fermentation

Numerous commercial microbial enzymes have been used to increase the functional value of phytochemicals present in plant sources however, lactic acid fermentation is preferred to improve the nutraceutical value of these foods because it is relatively inexpensive and improve overall organoleptic and nutritional characteristics (84). Contradictorily, PCs are able to exert an inhibitory effect on LAB (176). In addition, the incidence of certain chemical and physical parameters, such as the lack of fermentable carbohydrates, osmotic stress, and the acidic environment, are adverse conditions for bacterial growth. However, several LAB can adapt and grow in these substrates, being *L. plantarum* the most isolated species (166, 167, 177–179). The adaptation and survival strategies of LAB during cereal fermentation by activation of specific metabolic pathways have been investigated through a panel of various interacting omics approaches (metabolomic, phenomic, and transcriptomic profile) (180–183). The study of these adaptation responses would allow the optimal design of fermentation strategies for cereals and others plant matrices; however, these “omics” studies were not reported in pseudocereals fermentation.

**Figure 3:**
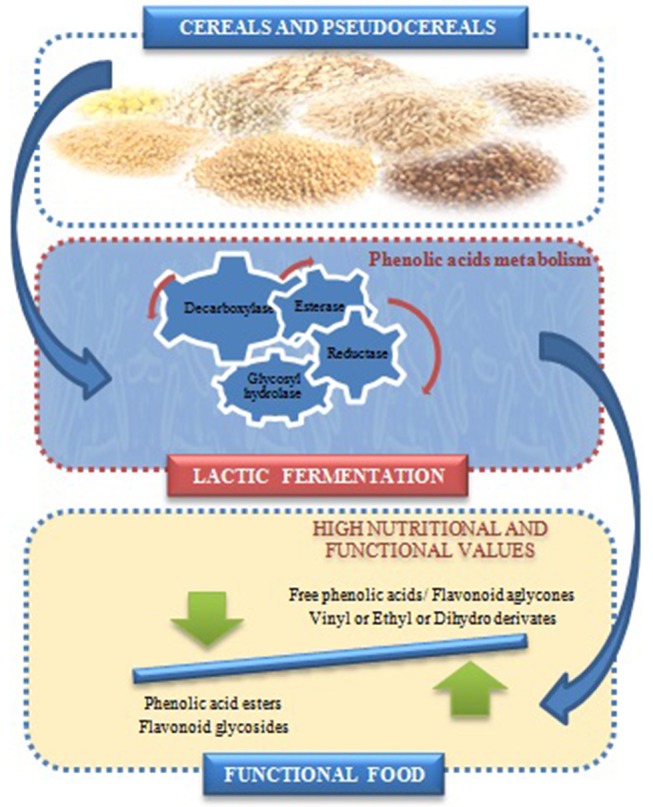
Effect of lactic acid fermentation on phenolic compounds profile of cereal and pseudocereals [adapted from (195)].

The effects of LAB on the release of PC and modification of phenolic profiles in both cereals and pseudocereals have been reported. They depend mainly on the grains types, species of microorganisms, fermentation conditions, particularly time, temperature, and pH values (159, 183–185). Some studies have highlighted the capacity of lactic fermentation of pseudocereals to enhance the PC in bread (159, 161, 186), beverages (173, 187, 188), tarhana soup (189) and pasta (190). The PC metabolism in LAB has two important physiological functions, it is an efficient mechanism to detoxify such compounds (191), and can have a role in the cellular energy balance because LAB employ hydroxycinnamic acids as external acceptors of electrons (192). The metabolism of PC by LAB was described principally in *L. plantarum* strains, and only few studies were reported in *Weissella* spp., *Leuconostoc mesenteroides, L. paracollinoides, L. hilgardii*, and *Oenococcus oeni* (192–194). The enzymes involved in the PC metabolism by LAB such as decarboxylases (PAD), reductases (PAR), esterases and/or glycosidases were reported [Figure 3; (176, 179, 196–198)]. The production of vinyl-phenol, vinyl-guaiacol and vinyl catechol from the *p*-coumaric, ferulic and caffeic acids, respectively, by PAD activities, are the most relevant (176, 177). Subsequently, these hydroxycinnamates by action of reductase are transformed to their corresponding phenylpropionic acids (199).

The pseudocereals (i.e., buckwheat and quinoa) have a higher content of flavonoids, mainly rutine, kaempferol, and quercetin, with respect to cereals such as rye and wheat (112, 200–204). The flavonoid aglycones are more potent in their functional action (i.e., antioxidant activity) than their corresponding glycosides. Shin et al. (205) showed that a strain of *Enterococcus avium* was able to metabolize rutine, a flavonol glycoside, in quercetin, a flavonol with many beneficial effects on health. Recently, Zielinski et al. (206) observed a decrease in rutin content in buckwheat flours fermented by different species of *Lactobacillus*. According to Yang et al. (207), quercetin has numerous biological and pharmacological effects, such as anticancer, antioxidative, antiviral, anti-inflammatory, and antiatherogenic activities. Fan et al. (208) reported the inhibition mechanism of quercetin on tyrosinase (rate-limiting enzyme in the melanogenesis pathway) and its potential use in the treatment of pigmentation disorders. In addition, Xiao (209) reviewed different biological benefits and pharmacokinetic behaviors between flavonoid glycosides and their aglycones. The elucidation of the metabolic pathways of these compounds will lead to obtain strains resistant to PC or adequate enzymes for cereals/pseudocereals processing and products with higher functional values, such as antioxidants.

#### Vitamins Produced by LAB

LAB and other vitamin-producing microorganisms can be used as an alternative to mandatory fortification in many countries to reduce deficiencies. Some LAB strains can produce elevated concentrations of the natural form of vitamins, which reduces the side-effects of chemically synthesized vitamins (masking of vitamin B_12_ deficiency, reduced enzyme activities in the liver, etc.) that are normally used (210, 211). Besides being a more natural alternative, vitamins producing LAB can also lower production costs by eliminating the need to add synthetic vitamins. The search for natural LAB strains from different ecological niches that can produce vitamins, such as folate, is essential in order to produce novel fermented foods that have high concentrations of this vitamin (212, 213). Vitamin producing strains have been able to revert and prevent vitamin deficiencies in animal models (52, 214).

LAB diversity is interesting not only at a species level, but also at the strain level, since most technological and nutritional properties are strain dependent. Raw cereals/pseudocereals constitute an interesting ecological niche to isolate new LAB strains with important characteristics to be used as a starter culture in the preparation of fermented cereal food. It was shown that folate producing LAB were isolated from wheat, sorghum and triticale (215). Previously, a few strains have been studied for this capacity in oat brans and rye sourdoughs (216, 217). In terms of vitamin-producing strains in pseudocereals, it has been shown that certain strains of LAB isolated from quinoa and amaranth sourdough have the capacity to produce elevated concentrations of riboflavin and folate in vitamin-free media (166, 167). These strains were used to obtain a B_9_ and B_2_ bio enriched pasta, which was able to prevent and revert vitamin deficiency in different rodent models (218).

In Africa, folate deficiency is related to the low dietary diversity and nutrient concentrations in complementary foods for infants (219, 220). In several African countries, cereal-based porridges are consumed as an alternative or in complement to breast feeding (221) but this product does not contain sufficient nutrients to prevent folate deficiencies (222). It has been suggested that porridges can also be consumed after fermentation with LAB, which can improve their overall nutritional quality, especially by increasing vitamin B_9_ concentrations (131, 223).

Several vitamin B_2_–producing LAB were isolated from durum wheat flour (224). Two *L. plantarum* over producer strains used as starter cultures were able to increase between 2 and 3 times the initial concentration of vitamin B_2_ in both, bread and pasta fermentations.

Russo et al. (225) reported that *L. fermentum* PBCC11 isolated from sourdough was able to produce riboflavin. Bread produced using the co-inoculum yeast and *L. fermentum* PBCC11.5 led to an approximately 2-fold increase of final vitamin B_2_ content compared to the wild-type strain (*L. fermentum* PBCC11). It was also reported that some LAB strains that are able to produce pseudo-vitamin B_12_ could also be used to increase the concentrations in cereal-based foods (226). These authors stated that the first pseudo-cobalamin producing strain of LAB was *L. reuteri* CRL 1098 that was isolated from sourdough (227). It has been suggested that the pseudo-cobalamin produced by LAB would not be biologically available since the intrinsic factor has a low affinity for this compound (228). However, it has been shown that soy fermented with this strain was able to prevent vitamin B_12_ deficiency in mice (229), demonstrating that pseudo cobalamins are bioavailable. The analysis of sequenced genomes of LAB will provide more insights and new potential candidates that could be used to ferment cereal-based foods.

#### Strategies to Decrease Phytates in Pseudocereals: Phytases

Different methods have been applied to reduce the PA content in grains and food to improve their nutritional value (230). Phytases, [myo-inositol (1–6) hexakisphosphate phosphohydrolase] constitute a particular subgroup of phosphatases capable of initiating the gradual dephosphorylation of phytate [myo-inositol (1–6) hexakisphosphate] forming myo-inositol phosphate intermediates decreasing or eliminating its antinutritional effect (231, 232). Phytases can be derived from different sources including plants, animals and microorganisms such as yeast and LAB; however, their structures are different (233).

One strategy to reduce phytate in cereals/pseudocereals includes treatments, such as soaking and malting (94) or germination (234, 235), that activate phytases present in plants (236). However, this activity is considered insufficient to eliminate the phytate present in these substrates (237). Recent research has shown that microbial sources are more promising for the production of phytases on a commercial level and in cereal based foods (238, 239).

**Figure 4:**
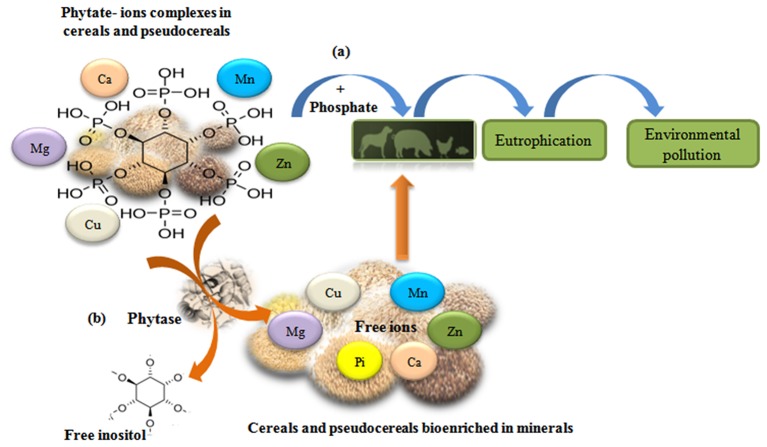
Phytate-ions complexes reduce bioavailability of minerals in cereal and pseudocereals. **(a)** Phosphorus supplementation in animal feedstuff cause pollution problems. **(b)** Phytase activity increase mineral biodisponibility and improve nutrition value of foods [adapted from (240)].

Nowadays, phytase is one of the most important enzymes for non-ruminant animal production. The application of phytases is broad, can be used to eliminate phytates in the food and feed industries, protect the environment by reducing phosphorus contamination and the eutrophication of water surfaces [Figure 4; (100, 239, 241, 242)]. Phytases have been successfully used in monogastric feeds for about to 20 years. In the beginning, marketable phytases were of fungal origin, mostly from *Aspergillus* species. Different studies have shown that the bioavailability of phosphorus increases, and the amount of phosphorus excreted is reduced (30–50%) by supplementing animal feeds with phytases (243–245). Recently, Theodoropoulos et al. (246) reported that treatment with commercial phytase decreased the content of myo-inositol phosphates and improved the nutritional value of soy drink, by improving the solubility of Ca^2+^, Fe^2+^, and Zn^2+^. Currently, the significance of bacterial phytases as potential tools in biotechnology is increasing (247).

##### LAB phytase activity

The prevalence of LAB in cereal ecosystems and their contribution to the improvement of this particular fermentation processes could be due to their biochemical and metabolic characteristics (248). Studies of enzymes, like phytases involved in nutritional aspects in determined ecosystems, are important for the understanding of particular traits of LAB that are relevant for their right exploitation as starters (249). The PA contained in gluten free flours can be reduced by lactic acid fermentation, directly by LAB phytase activity or indirectly providing the optimal conditions to the endogenous phytase activity (250). Several studies were carried out on different aspects of LAB phytases in cereals fermentation (129, 130, 163, 238, 251–253), nevertheless, there are only few reports of these enzymes in autochthonous LAB isolated from pseudocereals. Carrizo et al. (166, 167) reported high phytase activities in LAB strains isolated from quinoa and amaranth (grains and sourdough), such as *E. durans* CRL 2122 (1,041 ± 48 U/mL), *E. mundtii* CRL 2007 (957 ± 25 U/ml) and *L. plantarum* CRL 2106 (730 ± 25 U/mL), among other. Afterwards, the minerals bioavailability present in pasta made with quinoa flour as a dietary matrix and fermented by selected LAB producing phytase was evaluated in an animal model. The animal group fed with the bio-enriched pasta fermented by LAB (*L. plantarum* CRL 2107 + CRL 1964) showed higher concentrations of minerals (P, Ca^+2^, Fe^+2^, and Mg^+2^) with respect to control animal group (218). Also, Rizzello et al. (186) reported that the use of quinoa sourdough with autochthonous LAB (*L. plantarum* T6B10 and *L. rossiae* T0A16) increased phytase activity during the fermentation respect to non-fermented flour. The results confirmed that quinoa fermented with selected starters had a phytase activity ca. 2.75- times higher than raw quinoa flour.

Recent studies showed that phytate degradation by recombinant probiotic LAB could provide a solution for phosphate utilization in humans (254, 255). Regarding this topic, Vasudevan et al. (247) reviewed the contributions of recombinant technology to phytase research during the last decade with specific emphasis on new generation phytases. These results are relevant in the design of new functional foods with improved nutritional quality by using food-grade strains expressing microbial phytases.

## Conclusions

Throughout the world and especially in developing countries, the interest in pseudocereals has increased for both consumers and small businesses. Recent studies strongly suggest that non-essential nutrients like phytochemicals of pseudocereals can also have potential health beneficial effects. This fact has promoted different processing techniques that may enhance the biological value of pseudocereals. Despite the important nutritional and functional value of these grains, their commercialization is still quite limited. Lactic acid fermentation is an ancestral process of food preservation but with renewed interest over time. It has become an important strategy to exploit the bioactive potential of pseudocereals by hydrolysing anti-nutrients factors and increasing the level of health beneficial compounds. The multiple beneficial effects of pseudocereals fermented by selected LAB can be exploited in different ways leading to the design of novel plant-based foods that can target specific populations.

This review summarized recent research reporting some different beneficial effects of pseudocereals and contributes to increase the knowledge on LAB capacity to produce B-group vitamins, metabolize phytochemicals, and decrease phytates present in Andean grains. In this way, lactic acid fermentation can contribute to improve the nutritional and functional potential of fermented foods based on these grains for wide use throughout the world.

## Author Contributions

GR: writing—original draft, conceptualization, funding acquisition, and project administration. CG and JL: writing—original draft.

### Conflict of Interest Statement

The authors declare that the research was conducted in the absence of any commercial or financial relationships that could be construed as a potential conflict of interest.
